# Weight stigma and fat phobia in Poland – attitudes towards people living with obesity and the level of knowledge about obesity among the social media internet respondents and medical professionals

**DOI:** 10.3389/fnut.2023.1287783

**Published:** 2023-10-09

**Authors:** Karolina Świder, Alicja Baska, Mateusz Babicki, Agnieszka Mastalerz-Migas, Karolina Kłoda

**Affiliations:** ^1^NZOZ Biogenes, Wroclaw, Poland; ^2^Department of Lifestyle Medicine, School of Public Health, Centre of Postgraduate Medical Education, Warsaw, Poland; ^3^Polish Society of Lifestyle Medicine, Warsaw, Poland; ^4^Department of Family Medicine, Wroclaw Medical University, Wroclaw, Poland; ^5^Head of the Scientific Section of the Polish Society of Family Medicine, Wroclaw, Poland; ^6^MEDFIT Karolina Kłoda, Szczecin, Poland

**Keywords:** social media, fat phobia, fat phobia scale, FPS, obesity, stigma, healthcare professionals

## Abstract

**Introduction:**

Obesity often subjects individuals to stigmatization, impacting self-esteem, contributing to depression, social isolation, and even exacerbating weight gain. Our research aimed to evaluate weight stigma, fat phobia, their expressions, and obesity-related knowledge among social media internet respondents and medical practitioners in Poland.

**Methods:**

Conducted through Computer-Assisted Web Interview (CAWI), our study employed the Fat Phobia Scale (FPS) and tailored questions, analyzing 1705 questionnaires.

**Results:**

The respondents averaged a score of 3.60  ±  0.62 on the FPS. Interestingly, men exhibited higher stigma levels than women. Variables like BMI, residency, and interactions with people having obesity did not significantly impact stigma levels. Approximately 74.0% of respondents found individuals with obesity less attractive than those with normal weight, while 32.2% identified obesity as a cause of shame. Only 69.1% were aware of the BMI-based obesity diagnosis criterion.

**Conclusion:**

Given limited knowledge of Poland’s weight stigma landscape, our research yields crucial insights for shaping social campaigns and enhancing educational initiatives in obesity management for healthcare professionals. Further studies will be instrumental in addressing patient and practitioner needs effectively.

## 1. Introduction

World Health Organization (WHO) describes obesity and overweight as excessive or abnormal accumulation of fat tissue in the human body that may impair health and result from an energy imbalance between calories consumed and calories expended (1). Obesity in adults is identified when the calculated Body Mass Index (BMI), which is the ratio of weight to height, equals or exceeds 30 kg/m2. Overweight, on the other hand, is diagnosed when the BMI ranges from 25.0 to 29.9 kg/m2 (1, 2). Although BMI has its limitations and is not adequate for assessing both the degree of obesity and the potential health effects on the entire population, it remains the most commonly used indicator due to its ease of measurement and low cost (3, 4). Excess body weight is a global health and economic burden affecting people of all ages worldwide. According to global data, 39% of adults have excess body weight, defined as a BMI equal to or exceeding 25 kg/m2, and approximately 13% of them are diagnosed with obesity. Among children, around 18% have excess body weight (5). In the case of Poland, in 2022 the prevalence of overweight (BMI ≥ 25) among the Polish population aged 20 years or more was 62% for men and 43% for women, while obesity was 16 and 12%. Additionally, during the Covid-19 pandemic period from spring to autumn 2020, nearly 30% of Poles aged 20 years or older reported an increase in body weight (6).

Obesity has a tremendous impact on daily functioning and is the cause of over 200 health problems that worsen the quality of life and contribute to premature death (7). In addition to health damage, individuals with obesity are exposed to stigmatisation, which has been increasing in recent years (8). This also applies to other patient groups, especially those with mental disorders, where survey studies conducted in the general population and among medical students show that discrimination is a widespread phenomenon. Its negative consequences include low self-esteem, depression, social withdrawal, and in the case of people with obesity, even further weight gain (9–11). Moreover, it has been found that parents also demonstrate stigmatising attitudes towards their children with obesity, which can contribute to worsening their health, mainly in terms of mental well-being. This effect is considered the most significant threat resulting from obesity in children (12). Healthcare professionals also observe a negative attitude towards patients with excess body weight in their practice, stating at the same time that a patient living with obesity requires greater involvement, and may present greater challenges (13, 14). The topic has become significant enough to form an international expert group which, after gathering and analysing data, issued a statement recommending an end to the stigmatisation of people living with obesity (15). From a study conducted in Poland in 2019, it was found that individuals with obesity have a high level of knowledge about their condition (16). However, another study by the same authors indicated that 82.6% of the respondents experienced inappropriate behaviours. These behaviours were mostly reported from doctors (90%), nurses and midwives (51%), medical equipment operators (24%), dietitians (14%), and paramedics (9%). Specifically, 81% of the survey participants reported en-countering unpleasant and judgmental comments as the most common form of inappropriate behaviour they experienced (17).

To our knowledge the Fat Phobia Scale has not been used in the studies conducted in Poland so far. Moreover, the stigma level demonstrated by society and healthcare workers towards people living with obesity has not been adequately assessed. Additionally, Po-land is a country where the ‘Charter of Rights for Patients with Obesity’ was issued only this year. This document summarises the basic rights of patients living with obesity in their interactions with healthcare and institutions (18).

As representatives of the family medicine and lifestyle medicine community, who are active in social media, we are particularly interested in improving the perception, knowledge, and care for patients with obesity. In Poland, family doctors are the professionals who most frequently have contact with individuals with this medical condition. Primary healthcare, in particular, plays a crucial role in the care of patients with obesity, as it involves multidirectional actions in terms of prevention, diagnosis (including com-plications), treatment, and evaluation of referrals for specialised care (7). Therefore, as the Scientific Section of the Polish Society of Family Medicine in collaboration with Polish Society of Lifestyle Medicine, our study aimed to assess the phenomenon of stigmatisa-tion and its manifestations among social media internet respondents and medical professionals in Poland. Another objective was to understand the level of knowledge among Poles and healthcare workers regarding the causes, complications, and treatment methods of obesity. By obtaining information about attitudes towards individuals living with obesity and the knowledge possessed by the participants, we aim to contribute to effective strategies against stigmatisation and improve education in this area.

## 2. Methodology

### 2.1. Participants and recruitment

The study was conducted using a Computer-Assisted Web Interview (CAWI) and a customized questionnaire that was available to respondents online from April 25, 2023, to July 7, 2023. Before completing the questionnaire, participants were provided with in-formation about the research objectives and methodology and were required to provide informed consent. If consent was not given, the questionnaire could not be accessed. Throughout the survey, participants had the option to discontinue their participation without explanation. To ensure respondent anonymity, no personal data such as email addresses were collected.

The study’s inclusion criteria were being above 18 years old, residing in Poland, and having internet access. The survey was distributed anonymously through social media platforms like Facebook.com and Instagram, within various groups covering diverse topics, including those aimed at medical professionals. Additionally, study information was shared via email using the mailing lists of the Polish Society of Family Medicine and the Polish Society of Lifestyle Medicine.

The research utilized an online questionnaire prepared in Polish through Google Forms, comprising four sections. The first section collected demographic information (gender, age, place of residence) as well as professional and personal characteristics (e.g., weight, height, level of education, medical/non-medical profession). The respondents were also asked to evaluate the frequency of their contacts with people living with obe-sity. The second section employed the Fat Phobia Scale (FPS). The third section contained custom questions assessing the level of stigmatization, and the fourth section included custom questions related to knowledge about obesity.

### 2.2. The Fat phobia scale – short form

The FPS-short form is a standardised psychometric tool regarding beliefs and feel-ings towards people living with obesity. The scale comprises of 14 positive/negative ad-jectives aimed at characterising a person with obesity, using a five-point Likert scale. The final scale score is obtained by summing points from each question and dividing it by 14 (the number of scale items), resulting in a value ranging from 1 to 5. A higher score indicates a higher level of fat phobia. The internal consistency of the tool was found to be 0.893 (19).

For the purpose of this study, the original – English version of the FPS was translated to Polish. The Polish translation was blindly back-translated into English by a translator who had not seen the original version before. There were no significant differences between the translations.

### 2.3. Custom questions

The study questionnaire also included a series of customized questions designed to gather more specific information about the extent of weight stigma in Poland and to assess the level of participants’ knowledge about obesity.

It incorporated a multiple-choice question that asked participants to identify their feelings when encountering individuals with obesity (non-medical participants) or patients with obesity (medical professionals). These feelings included compassion, dislike, sympathy, impatience, friendliness, indifference, discomfort, contempt, willingness to help, and mercy.

Additionally, participants were asked to indicate their level of agreement with statements such as “obese individuals are inferior to those with normal body weight” and “obesity is a cause for shame.” Using a 10-point Likert scale, participants assessed their likelihood to hire a person with obesity, go on a date, form friendships, or entrust children in the care of someone with obesity. The Likert scale was also employed to evaluate respondents’ subjective assessment of the presence of weight discrimination in Poland.

The final section of the questionnaire explored participants’ level of knowledge about obesity. This part investigated the perception of obesity as a chronic disease and its associated complications, familiarity with the criteria for diagnosing obesity based on BMI, and awareness of effective treatment methods.

For a better understanding of the research methodology, the English version of the survey was attached in the Supplementary Table S1. The English Version of the Study Questionnaire.

### 2.4. Statistical analysis

The analysis was carried out using Statistica 13.0 by StatSoft (TIBCO Software, Palo Alto, CA, United States). The analysed variables were of qualitative and quantitative nature.

The normal distribution was assessed using the Shapiro–Wilk test. Basic descriptive statistics were ap-plied, including percentages, means, and standard deviations. The comparison of qualitative variables was conducted using the chi-square test. For quantitative variables, non-parametric tests such as the Mann–Whitney U test or Kruskal-Wallis Test were used. The degree of correlation between quantitative variables was evaluated using the Spearman correlation test. The significance level was assumed at 0.05.

## 3. Results

### 3.1. Characteristics of the study group

The study involved 1710 respondents, out of which 2 did not provide consent, and 3 respondents returned incomplete questionnaires. Therefore, the final analysis included 1705 completed surveys. The average age of the respondents was 34.7 ± 8.5 years. The majority were females (81.9%), residents of large cities (51.4%), and individuals with higher education (84.4%).Only a small number of respondents, 99 (5.8%), stated that they do not encounter individuals with obesity in their daily surroundings. Among the participants, 686 (40.2%) were healthcare sector professionals. A detailed overview of the characteristics of the study group is presented in Table 1.

**Table 1:** Characteristics of the Study Group, including comparison between medical and other respondents.

Analyzed variables		Studied group N(%) / M ± SD	Healthcare professional		
			Yes (N = 686) N(%) / M ± SD	No (N = 1,019) N(%) / M ± SD	*p*
Sex	Female	1,399 (81.9)	606 (88.0)	793 (77.8)	**<0.001** ^*^
	Male	298 (17.5)	81 (11.8)	217 (21.3)	
	Other	10 (0.6)	1 (0.2)	9 (0.9)	
Age [years]		34.7 ± 8.5	34.6 ± 7.7	34.8 ± 9.0	0.533^#^
Weight [kg]		73.5 ± 18.8	69.7 ± 16.8	76.1 ± 19.6	**<0.001** ^#^
Hight [cm]		169.4 ± 8.1	168.5 ± 7.4	170.1 ± 8.5	**<0.001** ^#^
BMI [kg/m^2^]		25.5 ± 5.9	24.8 ± 5.4	26.2 ± 6.2	**<0.001** ^#^
BMI	Underweight	63 (3.7)	35 (5.1)	28 (2.7)	**<0.001** ^*^
	Normal weight	911 (53.4)	416 (60.5)	495 (48.6)	
	Overweight	403 (23.6)	131 (19.0)	272 (26.7)	
	Obesity	330 (19.3)	106 (15.4)	224 (22.0)	
Place of residence	Rural area	195 (11.4)	69 (10.0)	126 (12.3)	**0.026** ^*^
	Town of up to 20,000 inhabitants	107 (6.3)	39 (5.7)	68 (6.7)	
	City of 20,000–100,000 inhabitants	216 (12.7)	99 (14.4)	117 (11.5)	
	City of 100,000–500,000 inhabitants	312 (18.2)	144 (20.9)	168 (16.5)	
	City of over 500,000 inhabitants	877 (51.4)	337 (49.0)	540 (53.0)	
Level of education	Higher	1,441 (84.4)	667 (97.0)	774 (76.0)	**<0.001** ^*^
	Secondary	237 (13.9)	18 (2.6)	219 (21.5)	
	Primary	8 (0.5)	---	8 (0.7)	
	Other	21 (1.2)	3 (0.4)	18 (1.8)	
Daily contact with a person living with obesity	Yes, often	1,006 (58.9)	535 (77.8)	471 (46.2)	**<0.001** ^*^
	Yes, rarely	602 (35.3)	141 (20.5)	461 (45.2)	
	No	99 (5.8)	12 (1.7)	87 (8.6)	

M – mean, SD – Standard deviation, N – numer, kg – kilograms, cm – centimeters, BMI – body mass index, # Mann–Whitney U test, * Chi-squared test.

Significant effects (<0.05) are marked in bold.

### 3.2. Fat phobia scale

In the analysis of the FPS scale (Table 2), respondents obtained an average score of 3.60 ± 0.62, which, in the case of the 14-item F-scale, could be seen as an indication of an average amount of fat phobia (19).

**Table 2:** Analysis of the FPS (Fat Phobia Scale) scores considering individual questions and distinguishing between medical and other respondents.

FPS pair of adjectives	Studied group M ± SD	Healthcare professional		
		Yes (N = 686) M ± SD	No (N = 1,019) M ± SD	*p* ^#^
Lazy/industrious	3.17 ± 0.90	3.16 ± 0.85	3.18 ± 0.94	0.609
No will power/has willpower	3.65 ± 0.91	3.63 ± 0.89	3.67 ± 0.97	0.214
Attractive/unattractive	3.68 ± 1.03	3.68 ± 0.94	3.68 ± 1.09	0.585
Good self-control/poor self-control	3.79 ± 0.90	3.81 ± 0.85	3.78 ± 0.94	0.645
Fast/slow	3.72 ± 0.98	3.67 ± 0.92	3.75 ± 1.01	**0.032**
Having endurance/having no endurance	3.47 ± 0.96	3.45 ± 0.91	3.49 ± 1.00	0.547
Active/inactive	3.58 ± 0.99	3.57 ± 0.95	3.59 ± 1.02	0.681
Weak/strong	3.02 ± 0.94	3.00 ± 0.86	3.03 ± 0.99	0.987
Self-indulgent/self-sacrificing	3.47 ± 1.00	3.42 ± 0.97	3.51 ± 1.01	0.059
Dislikes food/likes food	4.12 ± 0.89	4.13 ± 0.89	4.12 ± 0.89	0.821
Shapeless/shapely	3.43 ± 1.11	3.45 ± 1.04	3.42 ± 0.87	0.527
Undereats/overeats	4.07 ± 0.87	4.09 ± 0.87	4.06 ± 0.87	0.415
Insecure/secure	3.53 ± 1.01	3.62 ± 0.95	3.46 ± 1.03	**0.003**
Low-self-esteem/high self-esteem	3.69 ± 0.96	3.76 ± 0.92	3.64 ± 0.97	**0.012**
Final score	3.60 ± 0.62	3.60 ± 0.60	3.59 ± 0.66	0.883

Items 3, 4, 5, 6, 7, 10, and 12, scored as follows: 1 2 3 4 5.

Items 1, 2, 8, 9, 11, 13, and 14, score as follows: 5 4 3 2 1.

Fat Phobia Scale (FPS) score of the overweight vignette was from 1 = positive attributes to 5 = negative attributes.

M – mean, SD – Standard deviation, N – number.

#Mann–Whitney U test.

Significant effects (<0.05) are marked in bold.

When examining individual questions on the scale, the highest scores were obtained for the assessment of “dislikes food/likes food” - 4.12 ± 0.89 and “undereats/overeats” - 4.07 ± 0.87, while the lowest scores were for the comparisons “weak/strong” - 3.02 ± 0.94 and “lazy/industrious” - 3.17 ± 0.90. When assessing the influence of sociodemographic variables, it was found that men showed a higher level of stigmatisation than women (*p* < 0.001). However, no significant impact of responder’s BMI (*p* = 0.449), place of residence (*p* = 0.109), or daily contacts with individuals with obesity (*p* = 0.917) on the level of stigmatisation measured by FPS was observed. Similarly, no differences in an obtained average FPS score were found between medical and other respondents (3.60 ± 0.60 vs. 3.59 ± 0.66; *p* = 0.883).

A detailed analysis of the FPS scale scores is presented later, in Tables 3, 4.

**Table 3:** Analysis of the impact of demographic variables on the final score of the FPS (Fat Phobia Scale) and custom questions assessing the level of stigmatisation of patients with obesity.

Analyzed variables		FPS Final score		Probability of employment of a person with obesity		Probability of going on a date with a person with obesity		Probability of entrusting the care of your children to a person with obesity		Probability of befriending a person with obesity		People with obesity are discriminated in Poland	
		M ± SD	*p* ^#^	M ± SD	*p* ^#^	M ± SD	*p* ^#^	M ± SD	*p* ^#^	M ± SD	*p* ^#^	M ± SD	*p* ^#^
Sex	Female	3.58 ± 0.62	**<0.001**	8.32 ± 2.18	**<0.001**	4.71 ± 2.96	**<0.001**	7.96 ± 2.44	**<0.001**	8.89 ± 2.02	**<0.001**	7.84 ± 2.19	**<0.001**
	Male	3.73 ± 0.63		7.37 ± 2.51		3.04 ± 2.45		7.39 ± 2.56		8.11 ± 2.42		5.93 ± 2.77	
	Other	3.11 ± 0.44		9.7 ± 0.95		6.7 ± 3.74		9.4 ± 1.35		9.9 ± 1.64		9.3 ± 1.64	
BMI	Underweight	3.56 ± 0.60	0.449	7.96 ± 2.09	**0.004**	3.60 ± 2.51	**<0.001**	7.33 ± 2.33	**<0.001**	8.85 ± 1.99	**0.017**	7.78 ± 2.36	**<0.001**
	Normal weight	3.58 ± 0.58		8.07 ± 2.34		3.84 ± 2.82		7.77 ± 2.54		8.63 ± 2.10		7.25 ± 2.44	
	Overweight	3.63 ± 0.67		8.14 ± 2.17		4.52 ± 2.84		7.82 ± 2.37		8.86 ± 1.98		7.60 ± 2.33	
	Obesity	3.62 ± 0.68		8.49 ± 2.20		6.10 ± 2.90		8.34 ± 2.35		9.00 ± 2.07		8.07 ± 2.33	
Place of residence	Rural area	3.61 ± 0.65	0.109	8.16 ± 2.44	0.517	4.67 ± 2.94	**0.020**	7.86 ± 2.48	0.924	8.75 ± 2.23	0.101	7.65 ± 2.36	0.573
	Town of up to 20,000 inhabitants	3.51 ± 0.67		7.89 ± 2.37		5.14 ± 3.10		7.91 ± 2.35		8.75 ± 2.16		7.62 ± 2.37	
	City of 20,000–100,000 inhabitants	3.52 ± 0.70		8.18 ± 2.31		4.70 ± 2.97		7.84 ± 2.61		8.93 ± 2.05		7.54 ± 2.43	
	City of 100,000–500,000 inhabitants	3.57 ± 0.61		8.13 ± 2.34		4.52 ± 2.87		7.98 ± 2.42		8.85 ± 2.13		7.59 ± 2.43	
	City of over 500,000 inhabitants	3.64 ± 0.60		8.21 ± 2.18		4.19 ± 2.95		7.84 ± 2.46		8.69 ± 2.08		7.43 ± 2.42	
Level of education	Higher	3.62 ± 0.60	**0.005**	8.15 ± 2.24	0.113	4.35 ± 2.92	0.052	7.85 ± 2.45	0.191	8.78 ± 2.04	0.467	7.47 ± 2.42	**0.017**
	Secondary	3.45 ± 0.72		8.18 ± 2.46		4.82 ± 3.12		7.94 ± 2.61		8.57 ± 2.49		7.63 ± 2.40	
	Primary	3.63 ± 0.67		8.13 ± 2.64		6.00 ± 3.02		8.38 ± 2.77		8.63 ± 2.77		8.63 ± 1.18	
	Other	3.63 ± 0.75		9.05 ± 1.63		5.00 ± 2.68		8.72 ± 1.71		9.28 ± 2.00		8.81 ± 1.60	
Daily contact with a person living with obesity	Yes, often	3.60 ± 0.64	0.917	8.25 ± 2.23	0.151	4.83 ± 2.97	**<0.001**	7.99 ± 2.42	**0.006**	8.95 ± 1.96	**<0.001**	7.48 ± 2.45	0.626
	Yes, rarely	3.60 ± 0.60		8.02 ± 2.33		3.96 ± 2.81		7.67 ± 2.81		8.48 ± 2.29		7.52 ± 2.35	
	No	3.60 ± 0.66		8.15 ± 2.27		3.24 ± 2.85		7.87 ± 2.86		8.57 ± 2.30		7.67 ± 2.40	

M – mean, SD – Standard deviation, # Kruskal-Wallis Test, FPS - Fat Phobia Scale.

Significant effects (<0.05) are marked in bold.

**Table 4:** Correlation between the FPS score and the likelihood of employing a person with obesity, going on a date with them, entrusting children under their care, and befriending them.

FPS pair of adjectives	Probability							
	Employment of a person with obesity		Going on a date with a person with obesity		Entrusting the care of your children to a person with obesity		Befriending a person with obesity	
	*r*	*p*	*r*	*p*	*r*	*p*	*r*	*p*
Lazy/industrious	−0.202	<0.001	−0.214	<0.001	−0.203	<0.001	−0.176	<0.001
No will power/has willpower	−0.153	<0.001	−0.216	<0.001	−0.175	<0.001	−0.171	<0.001
Attractive/unattractive	−0.165	<0.001	−0.357	<0.001	−0.156	<0.001	−0.181	<0.001
Good self-control/poor self-control	−0.157	<0.001	−0.201	<0.001	−0.176	<0.001	−0.125	<0.001
Fast/slow	−0.120	<0.001	−0.181	<0.001	−0.129	<0.001	−0.100	<0.001
Having endurance/having no endurance	−0.178	<0.001	−0.179	<0.001	−0.187	<0.001	−0.140	<0.001
Active/inactive	−0.162	<0.001	−0.223	<0.001	−0.162	<0.001	−0.168	<0.001
Weak/strong	−0.161	<0.001	−0.181	<0.001	−0.190	<0.001	−0.178	<0.001
Self-indulgent/self-sacrificing	−0.204	<0.001	−0.172	<0.001	−0.203	<0.001	−0.201	<0.001
Dislikes food/likes food	−0.102	<0.001	−0.156	<0.001	−0.119	<0.001	−0.102	<0.001
Shapeless/shapely	−0.165	<0.001	−0.296	<0.001	−0.188	<0.001	−0.179	<0.001
Undereats/overeats	−0.134	<0.001	−0.222	<0.001	−0.159	<0.001	−0.131	<0.001
Insecure/secure	−0.019	0.427	−0.004	0.135	0.008	0.726	0.008	0.731
Low-self-esteem/high self-esteem	−0.021	0.374	−0.002	0.423	0.003	0.149	0.027	0.261
Final score	−0.215	<0.001	−0.318	<0.001	−0.222	<0.001	−0.208	<0.001

### 3.3. Custom questions assessing the level of stigmatization

In the analysis of questions evaluating how patients with obesity are perceived, it was found that 74% of respondents believe that individuals with obesity are less attractive than those with normal body weight, with 16.7% strongly agreeing with this statement. Additionally, on a 10-point scale, respondents rated the probability of going on a date with a person with obesity the lowest, with a score of 4.4 ± 3.0. A percentage of 8.4 of respondents considered individuals with obesity to be inferior to those with normal body weight, and 32.2% believed that obesity is a cause for shame. Among the respondents, the most common feelings accompanying interactions with people living with obesity included kindness (43.9%), willingness to help (34.0%), and compassion (33.4%). However, 8.4% of participants felt mercy, and 3.4% felt contempt in relation to contacts with indi-viduals with obesity.

When the impact of profession was analysed, it was observed that medical workers significantly less frequently agree with the statement that obesity is a cause for shame (29.9% vs. 33.7%; *p* < 0.031), although the percentage is still relatively high. Similar dif-ferences were found in the assessment that obese individuals are inferior to others (5.8% vs. 10.8%; *p* < 0.001). The most significant difference was seen in the willingness to help, where as many as 57.0% of medical workers declared such willingness compared to 18.6% of other respondents (*p* < 0.001).

According to the respondents’, patients with obesity in Poland face discrimination. This view is held by both medical and other respondents (*p* = 0.386).

A detailed overview of the questions related to obesity stigma is presented in Table 5, distinguishing between medical workers and other respondents.

**Table 5:** Custom questions assessing the level of stigmatisation of patients with obesity, distinguishing between medical workers and other respondents.

Analyzed Variables		Studied group N(%) / M ± SD	Healthcare professional		
			Yes (N = 686) N(%) / M ± SD	No (N = 1,019) N(%) / M ± SD	*p*
Are people with obesity worse than people with normal weight?	Definitely yes	20 (1.1)	5 (0.7)	15 (1.5)	**<0.001** ^*^
	Yes	30 (1.8)	11 (1.6)	19 (1.9)	
	Rather yes	94 (5.5)	24 (3.5)	70 (6.9)	
	Rather no	240 (14.1)	78 (11.3)	162 (15.9)	
	No	240 (31.4)	231 (33.6)	305 (29.9)	
	Definitely no	787 (46.1)	339 (49.3)	448 (44.0)	
Are people with obesity less attractive than people with normal weight?	Definitely yes	285 (16.7)	99 (14.4)	186 (18.3)	0.085^*^
	Yes	363 (21.3)	141 (20.5)	222 (21.8)	
	Rather yes	615 (36.0)	273 (39.7)	342 (33.6)	
	Rather no	213 (12.5)	83 (12.1)	130 (12.8)	
	No	138 (8.1)	59 (8.6)	79 (7.8)	
	Definitely no	93 (5.4)	33 (4.8)	60 (5.9)	
Would you hire a person with obesity as an employer?	Definitely yes	485 (28.4)	175 (25.4)	310 (30.4)	0.262^*^
	Yes	721 (42.2)	312 (45.4)	309 (40.1)	
	Rather yes	374 (21.9)	151 (22.0)	223 (21.9)	
	Rather no	20 (1.2)	38 (5.5)	57 (5.6)	
	No	95 (5.6)	7 (1.0)	13 (1.3)	
	Definitely no	12 (0.7)	5 (0.7)	7 (0.7)	
Is obesity a cause for shame?	Definitely yes	51 (3.0)	14 (2.0)	37 (3.6)	**0.031** ^*^
	Yes	377 (22.1)	36 (5.2)	86 (8.4)	
	Rather yes	122 (7.1)	156 (22.7)	221 (21.7)	
	Rather no	419 (24.5)	195 (28.3)	286 (28.1)	
	No	481 (28.2)	185 (26.9)	234 (23.0)	
	Definitely no	257 (15.1)	102 (14.8)	155 (15.2)	
On a scale of 1 to 10, how likely is it in your case?					
Employment of a person with obesity		8.2 ± 2.3	8.1 ± 2.3	8.2 ± 2.3	0.401^#^
Going on a date with a person with obesity		4.4 ± 3.0	4.5 ± 2.9	4.4 ± 3.0	0.402^#^
Entrusting the care of your children to a person with obesity		7.9 ± 2.5	7.9 ± 2.4	7.9 ± 2.5	0.909^#^
Befriending a person with obesity		8.8 ± 2.1	8.9 ± 1.9	8.7 ± 2.2	**0.012** ^#^
People with obesity are discriminated in Poland		7.5 ± 2.4	7.6 ± 2.3	7.4 ± 2.5	0.386^#^
Perceptions in contact with an obese person					
Mercy		143 (8.4)	59 (8.6)	84 (8.2)	0.808^#^
Reluctance		186 (10.9)	60 (8.7)	126 (12.4)	**0.018** ^**#**^
Contempt		64 (3.7)	18 (2.6)	46 (4.4)	0.053^#^
Kindness		749 (43.9)	326 (47.4)	423 (41.5)	**0.016** ^**#**^
Discomfort in contacts		245 (14.3)	85 (12.4)	160 (15.7)	0.053^#^
Sympathy		460 (26.9)	162 (23.6)	298 (29.2)	**0.009** ^**#**^
Impatience		128 (7.5)	71 (10.3)	57 (5.5)	**<0.001** ^**#**^
Indifference		534 (31.3)	133 (19.3)	401 (39.3)	**<0.001** ^**#**^
Compassion		570 (33.4)	260 (37.8)	310 (30.4)	**0.001** ^**#**^
Willingness to help		581 (34.0)	392 (57.0)	189 (18.6)	**<0.001** ^**#**^

M – mean, SD – Standard deviation, N – numer, # Mann–Whitney U test, * Chi-squared test.

Significant effects (<0.05) are marked in bold.

In the sociodemographic variables analysis, in the question regarding the likelihood of dating someone with obesity, men obtained a score 1.67 points lower (*p* < 0.001) than women. Men were less likely to befriend someone with obesity (*p* < 0.001), employ them (*p* < 0.001), or even entrust their children under their care (*p* < 0.001). On a 10-point Likert scale assessing whether individuals with obesity are discriminated against, men had an average score of 5.93 ± 2.77 points, while women had 7.84 ± 2.19 points (*p* < 0.001).

In the analysis of the impact of respondent’s BMI on the perception of discrimination against people with obesity in Poland, it was found that individuals meeting the obesity criteria obtained the highest scores. Similarly, they also obtained the highest scores in as-sessing the likelihood of dating, befriending, employing, or providing childcare. Detailed results are presented in Table 3.

It was also shown that there is a negative correlation between the FPS score and the questions regarding the likelihood of employing a person with obesity (r = −0.215, *p* < 0.001), going on a date with them (*r* = −0.318, *p* < 0.001), entrusting children under their care (r = −0.222, *p* < 0.001), and befriending them (*r* = −0.208, *p* < 0.001). A detailed overview is presented in Table 4, including the individual FPS questions.

### 3.4. Level of knowledge about obesity

In the analysis of questions aimed at assessing the level of knowledge about obesity, it was found that only 69.1% of respondents are aware of the criteria for diagnosing obesity based on BMI. A percentage of 94.1 of respondents correctly indicated that obesity is a chronic disease that requires treatment, but 1.5% believe that obesity is not a disease. Among the reasons for the development of obesity, respondents most commonly pointed out excessive calorie intake (96.5%) and complications from certain medications (88.5%). Interestingly, 50.2% of respondents incorrectly identified hyperthyroidism as a cause of obesity. The most commonly indicated effective treatment methods were regular physical activity (95.4%) and pharmacological treatment (78.3%). Surprisingly, 4.2% respondents considered fasting as an effective form of obesity treatment.

Among most commonly indicated obesity-related complications, diabetes (96.3%), hypertension (95.7%), and decreased exercise tolerance (95.2%) were chosen. Only 49.9% of respondents indicated that obesity can contribute to the development of dementia.

Comparison between medical professionals with other respondents showed a higher level of knowledge among healthcare workers. Nevertheless, 15.3% of medical professionals were not familiar with the BMI criteria for diagnosing obesity, 26.5% believed that hyperthyroidism causes obesity, and 4.1% considered fasting as an effective form of obesity treatment. A detailed overview of the level of knowledge, distinguishing between healthcare professionals and other respondents, is presented in Table 6.

**Table 6:** Assessment of knowledge level regarding obesity, distinguishing between medical workers and other respondents.

Variables		Studied group N(%)	Healthcare professional		
			Yes (N = 686) N(%)	No (N = 1,019) N(%)	p*
Obesity	is a chronic disease that requires treatment	1,607 (94.1)	678 (98.6)	929 (91.1)	**<0.001**
	is a disease that does not require treatment	76 (4.4)	2 (0.3)	22 (2.2)	
	is not a chronic disease	25 (1.5)	8 (1.1)	68 (6.7)	
BMI criterion for the diagnosis of obesity	≥ 25	146 (8.5)	41 (6.0)	104 (10.2)	**<0.001**
	≥ 27.5	223 (13.1)	39 (5.7)	184 (18.0)	
	≥ 30	1,181 (69.1)	583 (84.7)	598 (58.7)	
	≥ 29	159 (9.3)	25 (3.6)	133 (13.1)	
Causes of obesity	Lack of physical activity	1,510 (88.4)	649 (94.3)	861 (84.5)	**<0.001**
	Excessive calorie supply	1,649 (96.5)	680 (98.8)	968 (95.0)	**<0.001**
	Certain chronic diseases, e.g., diabetes	1,362 (79.7)	494 (71.8)	868 (85.2)	**<0.001**
	Hyperthyroidism	858 (50.2)	182 (26.5)	676 (66.3)	**<0.001**
	Effects of certain drugs, e.g., stereoids, antipsychotics	1,512 (88.5)	626 (91.0)	886 (87.0)	**0.011**
Effective form of obesity treatment	Bariatric surgery	1,309 (76.6)	589 (85.6)	719 (70.6)	**<0.001**
	Pharmacological treatment	1,337 (78.3)	599 (87.1)	738 (72.4)	**<0.001**
	Regular physical activity	1,630 (95.4)	671 (97.5)	959 (94.1)	**<0.001**
	Starvation	71 (4.2)	28 (4.1)	43 (4.2)	0.879
Obesity complications	Poorer exercise tolerance/fatigue	1,626 (95.2)	679 (98.7)	947 (92.9)	**<0.001**
	Hypertension	1,634 (95.7)	679 (98.7)	954 (93.6)	**<0.001**
	Diabetes mellitus	1,645 (96.3)	677 (98.4)	968 (95.0)	**<0.001**
	Hormonal disorders	1,552 (90.9)	660 (95.9)	892 (87.5)	**<0.001**
	Female menstrual disorders	1,398 (81.9)	639 (92.9)	759 (74.5)	**<0.001**
	Decrease in libido	1,441 (84.4)	641 (93.2)	800 (78.5)	**<0.001**
	Deterioration of hair and/or nails growth	1,119 (65.5)	514 (74.7)	605 (59.4)	**<0.001**
	Depression	1,617 (94.7)	677 (98.4)	940 (92.3)	**<0.001**
	Future dementia	853 (49.9)	437 (63.5)	416 (40.8)	**<0.001**
	Gout	901 (52.8)	493 (71.7)	408 (40.0)	**<0.001**

Significant effects (<0.05) are marked in bold, * Chi-squared test.

## 4. Discussion

This study was conducted to assess the current attitudes of social media users and healthcare workers in Poland towards individuals living with obesity, as well as to evaluate knowledge about this disease. Analysing the Fat Phobia Scale (FPS), the average score was found to be 3.60 ± 0.62 (3.59 for medical professionals), with significant differ-ences in responses for fast/slow, insecure/secure, and low-self-esteem/high self-esteem questions. The analysis of sociodemographic variables revealed that men demonstrate a higher level of stigmatisation than women (*p* < 0.001), and BMI (*p* = 0.449) or daily contacts with individuals with obesity (*p* = 0.917) did not have an impact on the level of stigmatisation measured by FPS.

Two Mexican studies from 2014 and 2015, analysing FPS scores among medical and psychological (20), as well as nutrition students (21), reported average scores of 3.40 and 3.45, respectively. Interestingly, similar to our findings, in the first study, men exhibited significantly higher levels of fat phobia, and in the second study, no association was found between BMI and daily contacts with individuals with obesity. Conclusions from both studies highlighted the prevalence of moderate to high levels of stigmatisation and the need for preventive actions to address fat phobia. A 2021 study conducted in Turkey among outpatient clinic patients using FPS indicated an average score of 3.07 for both men and women. The level of stigmatisation was not associated with respondents’ BMI. The authors emphasised that overcoming prejudice can be achieved through education. While fat phobia exists at various levels, minimising it requires identifying situations of its occurrence and increasing awareness (22).

Two Polish publications from 2021 and 2023 also addressed attitudes towards the discrimination of patients with obesity (23) and the level of knowledge about obesity among medical professionals (24). They were based on a custom questionnaire and in-volved 184 medical professionals. Findings from the first study indicated that the majority of medical professionals (68.5%) considered worse attitudes towards patients with obesity to be a common phenomenon. About half of them (48.4%) witnessed discriminatory behaviours among medical staff. The most common forms of inappropriate behaviour included making jokes about appearance (96.6%), looks of disgust and aversion (96.2%), and not reacting to offensive remarks (92.0%). Participants also highlighted systemic dis-criminatory constraints, such as limited access to dedicated medical equipment (62.4%) (23). The second study assessed the knowledge about obesity. Percentage of 67.1 of respondents provided correct answers, with better accuracy in regard to obesity diagnosis (70.1%) than treatment methods (64.6%) (24). However, neither of these studies utilised FPS or any other standardised tool, nor included direct questions evaluating stigmatisation reported by respondents. An earlier Polish study, published in 2016 used the Anti-fat Attitudes Scale (AFAS), but not validated then. This is a short tool, which measures negative attitudes toward individuals with excessive weight. It was indicated that educational intervention is effective in reducing weight stigma (25). The validation of AFAS was carried out in 2018 (26).

Due to the lack of relevant Polish studies for comparison, we selected a study conducted in Germany - neighbouring Poland, published in 2014, and involving a simi-lar-sized population (1,657 subjects). The authors reported an average FPS score of 3.62, which was very close to our analysis and was dependent on body weight and age. Younger individuals with lower BMI exhibited higher levels of stigmatisation. One conclusion of this study suggests that the abbreviated FPS could be used more extensively to assess the effectiveness of campaigns dedicated to combating fat phobia or various in-terventions to reduce stigmatisation towards individuals with obesity (27). A Swedish study from 2022 that assessed attitudes towards obesity used a custom questionnaire based on surveys from other countries and analysed responses of 235 primary care physicians (the questionnaire was sent to 1,642 GPs). Nearly half of the respondents (47%) believed that a lack of self-control is one of the causes of obesity, 22% attributed obesity to laziness, and 14% stated that individuals with obesity lack motivation to lose weight. Alongside evaluating physicians’ attitudes, the authors also assessed their knowledge. A high percentage (91%) of correct answers were obtained, indicating that obesity is recognized as a disease. Surprisingly, more than half of the respondents (58%) did not believe that medications used in obesity treatment are effective for weight reduction in individ-uals with a BMI between 30 and 39.9 kg/m2, and 20% were unsure about it. Hence, the vast majority of physicians did not acknowledge the supportive role of pharmacological obesity treatment (28). In our research, we noticed the percentage of respondents who were unaware of the obesity diagnosis criteria (30.9%), among which 15.3% were medical professionals. Additionally, a significant proportion (50.2%) of respondents wrongly identified hyperthyroidism as a cause of obesity, including 26.5% of healthcare workers.

Because of the deficiency of dedicated tools in Poland to assess the level of stigmatisation, we decided to create custom questions. In the case of other countries, there are some useful tools to assess weight stigma and body dissatisfaction, like Modified Weight Bias Internalization Scale (WBIS-M), a modified Body Parts Satisfaction Scale (BPSS), Korean version of the Weight Self-Stigma Questionnaire (WSSQ-K) or Anti-Fat Attitudes (AFA) questionnaire and Beliefs about Obese People (BAOP) (29–31). To the authors’ best knowledge, this research is one of the first and largest studies dedicated to this subject in Poland. So far, only a report titled “Vingardium Grubiosa” has been published, focusing on the experiences of individuals living with obesity with healthcare system (32). However, it does not have a scientific nature and is not available in English. Thus, in order to achieve the objectives of reducing and eliminating weight stigma, we need more evidence-based research. In particular, to form the basis for positions such as the Charter of Rights for Patients with Obesity, developed with the involvement of the Polish Society for Obesity Treatment, is a comprehensive document outlining the fundamental rights of individuals living with obesity. These rights encompass, among others, access to healthcare and life, respect for personal dignity and the demonstration of respect, the right to self-determination, and the prevention of violence, inhumane, and humiliating behaviour. This document represents a groundbreaking initiative in Poland, designed to shed light on the available opportunities, based on current legal regulations, for providing medical care to people with obesity in line with modern medical knowledge. It also strives to ensure equal access to reliable information, proper diagnosis, and treatment, all within conditions conducive to enhancing health (18).

The use of social media in our study allowed us to disseminate the survey faster and gave us the opportunity to reach out to higher amount of respondents. Especially that Facebook.com not only facilitates creating private medical topic groups but also gives possibility to take part in generally available thematic conversations. A study from United States analyzed the COVID-19 pandemic obesity-related discourse on Facebook and Instagram. When it became known that obesity worsens the prognosis of SARS-CoV-2 infection, numerous media information on this subject emerged, but also an increase in weight stigma occurred. This was reflected in more obesity-related posts on both evalu-ated social media platforms. However, the discussed topics were not always medically accurate. Although topics related to diet were more often posted on Instagram, “clickbait” was more prevalent on Facebook (33). Social media also plays a huge role in creating vi-sions of the ideal figure and can foster feelings of inadequacy among its users. As a result, media, including social media, can lead to an increased sense of stigmatisation of people with obesity and lead to a number of negative consequences including, but not limited to, eating disorders (34). An interesting Polish-Spanish study examining the use of social media among gay man with orthorexia nervosa has been published just recently. Authors found that use of the Grindr application was a significant predictor of orthorexia nervosa, whereas use of Instagram decreased the risk. Grindr usage is associated with body image distortion and feelings of sexual objectification, weight stigma, and social comparisons (35).

Our study showed that individuals with obesity are perceived as inferior and less attractive (74% of respondents) and would be invited on a date with the lowest probability, with 32.2% of participants considering obesity as a cause for shame. Although medical professionals significantly less often agree with this statement, still as many as 29.9% of them hold such a belief. At the same time, respondents unanimously notice that individuals with obesity are discriminated against in Poland. Significant differences were observed in the declarations of attitudes towards people with obesity between medical professionals and other respondents. The most significant difference was found in the willingness to help, where as many as 57.0% of medical professionals made such a declaration compared to 18.6% of other respondents. The analysis of the impact of demographic variables on FPS scores revealed differences between genders, with men showing a higher level of stigmatisation. Individuals meeting the BMI criteria for obesity obtained the highest score in the question assessing the discrimination of patients with obesity in Poland. On the other hand, the correlation analysis revealed an inverse relationship between the FPS score and the likelihood of employment, going on a date, leaving children under the care of, and making friends with a person with obesity.

Our findings present a certain segment of attitudes that can be subject to generalisation. A publication from Ireland in 2018, co-authored by Carel Le Roux, well-known for advocating on behalf of individuals living with obesity, raises a challenging question - “How Ethical Is Our Current Delivery of Care to Patients with Severe and Complicated Obesity?” The number of patients with severe obesity (BMI > 40 kg/m2) is rapidly increasing. Despite bariatric surgery being an effective and cost-effective treatment, many patients do not opt for surgical consultations due to previous unpleasant experiences with healthcare professionals. Widespread biases and stigma imposed on individuals with obesity perpetuate a lack of action in terms of social and health policy support. Therefore, the conclusion of the study is to establish prevention of harm and ensure fair and transparent allocation of resources for all healthcare decision-makers when considering the care of individuals with severe obesity (36).

The authors are aware of the limitations of this study, particularly related to data collection methods. Nevertheless, conducting the survey via the internet and social media provided access to a wide audience from all over Poland. Furthermore, previous publications have shown that respondents tend to provide more honest answers in anonymous internet surveys. Internet-based surveys have also been found to reduce respondent anxiety, thereby increasing the likelihood of their participation in the study (37). Another methodological limitation is the inability to estimate the response rate. It is also important to note that the analysed sample is not representative of the Polish population or healthcare workers. Therefore, further studies are necessary on a representative group of individuals.

In summary, in Poland, both among the social medial users population and healthcare workers, a medium-to-high level of fat phobia and high level of stigmatisation can be observed, particularly among men. At the same time, the respondents demonstrated a relatively low level of knowledge, regarding the diagnostic criteria of obesity and causes of obesity, also among healthcare workers. Compared to other countries, Poland exhibits both educational gaps regarding obesity as a disease and a less favourable attitude towards individuals living with obesity. To address these issues among policymakers, the interdisciplinary Partnership for Obesity Prevention and Treatment was es-tablished in December 2022, initiated by the Polish Society for Obesity Treatment and the Institute of Health Management at Lazarski University. The Partnership aims to identify areas where key activities should be undertaken based on reliable and comprehensive information reaching those responsible for shaping the healthcare and education systems (38). We hope that the results of our study will be helpful for further activities in this area.

## 5. Conclusion

The situation of patients with obesity in Poland requires improvement. Due to the medium-to-high level of fat phobia and high level of stigmatisation, as well as relatively low knowledge about obesity both among the social media users and healthcare workers, it is justified to introduce social informational campaigns aimed at countering stigmatization. Moreover, enhancing and expanding educational offerings and better reaching healthcare professionals with such programs focused on recognizing and treating obesity are essential. Another challenge is to engage policy-makers in the topic of obesity, where organised patient groups and experts can provide assistance. Our study provided new information about the extent of weight stigma in Poland, but further research and reports in this area are needed to better address the needs of both patients and healthcare professionals.

## Data availability statement

The raw data supporting the conclusions of this article will be made available by the authors, without undue reservation.

## Ethics statement

The studies involving humans were conducted in accordance with the Declaration of Helsinki and were approved by the Bioethics Committee of the Wroclaw Medical University, Poland. The studies were conducted in accordance with the local legislation and institutional requirements. Written informed consent for participation was not required from the participants or the participants legal guardians/next of kin in accordance with the national legislation and institutional requirements.

## Author contributions

KŚ: Conceptualization, Software, Writing – original draft, Writing – review & editing, Methodology, Validation. AB: Funding acquisition, Methodology, Resources, Writing – original draft, Writing – review & editing. MB: Conceptualization, Formal analysis, Investigation, Methodology, Project administration, Supervision, Writing – original draft, Writing – review & editing. AM-M: Funding acquisition, Supervision, Writing – original draft, Writing – review & editing. KK: Conceptualization, Investigation, Methodology, Project administration, Validation, Visualization, Writing – original draft, Writing – review & editing.
