# Supplementary material for: Weight stigma and fat phobia in Poland – attitudes towards people living with obesity and the level of knowledge about obesity among the social media internet respondents and medical professionals
Source: Front Nutr. 2023 Oct 9;10:1287783. doi: 10.3389/fnut.2023.1287783 (PMC10593449; doi:10.3389/fnut.2023.1287783)
Supplement: Supplementary file 1 [file Data_Sheet_1.PDF]

frontiers

Supplementary Material

Table S1. The English Version of the Study Questionnaire

Demographic variables	
Sex	Female
	Male
	Other
How old are you? [years]	
Your place of residence	Rural area
	Town up to 20,000 inhabitants
	City of 20,000-100,000 inhabitants
	City of 100,000-500,000 inhabitants
	City of over 500,000 inhabitants
Your level of education	Primary
	Secondary
	Higher
	Other
Your weight [kg]	
Your height [cm]	
Are you a healthcare professional?	Yes

	No
General questions after choosing „No” in Q: Are you a healthcare professional?	
Do you have contact with people living with obesity daily?	Yes, often
	Yes, rarely
	No
What kind of feelings do you experience when interacting with a person with obesity?	Mercy
	Reluctance
	Contempt
	Kindness
	Discomfort
	Sympathy
	Impatience
	Indifference
	Compassion
	Willingness to help
Additional questions after choosing „Yes” in Q: Are you a healthcare professional?	
What profession are you in? *(alphabetical order)*	Dietitian
	Medical caregiver
	Nurse
	Paramedic

<table>
<tr><td rowspan="7"></td><td colspan="2">Pharmacist</td></tr>
<tr><td rowspan="3">Physician;
if yes:
- What specialty??</td><td>Surgical</td></tr>
<tr><td>Non-surgical</td></tr>
<tr><td>Family medicine</td></tr>
<tr><td></td><td></td></tr>
<tr><td colspan="2">Physiotherapist</td></tr>
<tr><td colspan="2">Other than listed</td></tr>
<tr><td>How many years have you worked in the profession? [years]</td><td colspan="2"></td></tr>
<tr><td rowspan="4">Your main workplace</td><td colspan="2">Hospital</td></tr>
<tr><td colspan="2">Specialist clinic</td></tr>
<tr><td colspan="2">Primary care clinic</td></tr>
<tr><td colspan="2">Private practice</td></tr>
<tr><td rowspan="3">Do you encounter patients with obesity in your professional role?</td><td colspan="2">Yes, often</td></tr>
<tr><td colspan="2">Yes, rarely</td></tr>
<tr><td colspan="2">No</td></tr>
<tr><td rowspan="5">What kind of feelings do you experience when interacting with a patient with obesity?</td><td colspan="2">Mercy</td></tr>
<tr><td colspan="2">Reluctance</td></tr>
<tr><td colspan="2">Contempt</td></tr>
<tr><td colspan="2">Kindness</td></tr>
<tr><td colspan="2">Discomfort</td></tr>
</table>

	Sympathy
	Impatience
	Indifference
	Compassion
	Willingness to help
Is taking care of a patient with obesity more demanding than caring for a patient with a normal body weight (e.g., more challenging to perform medical procedures, technical difficulties during patient examination)?	Yes
	No
Questions to every responder	
Fat Phobia Scale	
Listed below are 14 pairs of adjectives sometimes used to describe obese or fat people. For each adjective pair, please place an X on the numer closest to the adjective that you feel best describes your feelings and beliefs.	

Lazy	1 – 2 – 3 – 4 - 5	Industrious
No will power	1 – 2 – 3 – 4 – 5	Has willpower
Attractive	1 – 2 – 3 – 4 – 5	Unattractive
Good self-control	1 – 2 – 3 – 4 – 5	Poor self-control
Fast	1 – 2 – 3 – 4 – 5	Slow
Having endurance	1 – 2 – 3 – 4 – 5	Having no endurance
Active	1 – 2 – 3 – 4 – 5	Inactive
Weak	1 – 2 – 3 – 4 – 5	Strong
Self-indulgent	1 – 2 – 3 – 4 – 5	Self-sacrificing

<table>
<tr><td>Dislikes food</td><td colspan="2">1 – 2 – 3 – 4 – 5</td><td>Likes food</td></tr>
<tr><td>Shapeless</td><td colspan="2">1 – 2 – 3 – 4 – 5</td><td>Shapely</td></tr>
<tr><td>Undereats</td><td colspan="2">1 – 2 – 3 – 4 – 5</td><td>Overeats</td></tr>
<tr><td>Insecure</td><td colspan="2">1 – 2 – 3 – 4 – 5</td><td>Secure</td></tr>
<tr><td>Low-self-esteem</td><td colspan="2">1 – 2 – 3 – 4 - 5</td><td>High self-esteem</td></tr>
<tr><td colspan="4">Custom questions assessing the level of stigmatisation.</td></tr>
<tr><td colspan="3" rowspan="6">Are people with obesity worse than people with normal weight?</td><td>Definitely yes</td></tr>
<tr><td>Yes</td></tr>
<tr><td>Rather yes</td></tr>
<tr><td>Rather no</td></tr>
<tr><td>No</td></tr>
<tr><td>Definitely no</td></tr>
<tr><td colspan="3" rowspan="6">Are people with obesity less attractive than people with normal weight?</td><td>Definitely yes</td></tr>
<tr><td>Yes</td></tr>
<tr><td>Rather yes</td></tr>
<tr><td>Rather no</td></tr>
<tr><td>No</td></tr>
<tr><td>Definitely no</td></tr>
<tr><td colspan="3" rowspan="2">Would you hire a person with obesity as an employer?</td><td>Definitely yes</td></tr>
<tr><td>Yes</td></tr>
</table>

	Rather yes
	Rather no
	No
	Definitely no
Is obesity a cause for shame?	Definitely yes
	Yes
	Rather yes
	Rather no
	No
	Definitely no
On a scale of 1 to 10, how likely would you be to employ a person with obesity?	
On a scale of 1 to 10, how likely would you be to go out on a date with a person with obesity?	
On a scale of 1 to 10, how likely would you be to entrust the care of your children to a person with obesity?	
On a scale of 1 to 10, how likely would you be to befriend a person with obesity?	
On a scale of 1 to 10, how much do you rate the level of discrimination against people with obesity in Poland	
Questions regarding level of knowledge about obesity	
In your opinion, obesity is:	a chronic disease that requires treatment

	a disease that does not require treatment
	not a chronic disease
The criteria for diagnosing obesity using BMI is:	≥ 25
	≥ 27.5
	≥ 30
	≥ 29
In your opinion, what could be the causes of obesity? (possible more than one answer)	Lack of physical activity
	Excessive calorie supply
	Certain chronic diseases, e.g. diabetes
	Hyperthyroidism
	Effects of certain drugs e.g. stereoids, antipsychotics
An effective form of obesity treatment is: (possible more than one answer):	Bariatric surgery
	Pharmacological treatment
	Regular physical activity
	Fasting
According to you, can obesity be a cause of: (possible more than one answer)	Poorer exercise tolerance/fatigue
	Hypertension
	Diabetes mellitus
	Hormonal disorders

	Female menstrual disorders
	Decrease in libido
	Deterioration of hair and/or nails growth
	Depression
	Future dementia
	Gout

Table S2. **The Polish Version of the Study Questionnaire**

Zmienne demograficzne	
Płeć	Kobieta
	Mężczyzna
	Inna
Ile ma Pan(i) lat? [lata]	
Miejsce zamieszkania:	Wieś
	Miasto do 20 000 mieszkańców
	Miasto od 20 000 do 100 000 mieszkańców
	Miasto do 100 000 do 500 000 mieszkańców
	Miasto powyżej 500 000 mieszkańców

Wykształcenie	Podstawowe
	Średnie
	Wyższe
	Inne niż wymienione
Waga [kg]	
Wzrost [cm]	
Czy jest Pan(i) profesjonalist(k)ą ochrony zdrowia?	Tak
	Nie
Pytania ogólne po wybraniu opcji "Nie" w pytaniu Q: Czy jest Pan(i) profesjonalist(k)ą ochrony zdrowia?	
Czy jest Pan(i) profesjonalist(k)ą ochrony zdrowia?Czy na co dzień w swoim otoczeniu ma Pan(i) kontakt z osobami z otyłością?	Tak, często
	Tak, czasami
	Nie
Jakich odczuć doznaje Pan(i) podczas kontaktu z osobą z otyłością?	Litość
	Niechęć
	Pogarda
	Życzliwość
	Dyskomfort w kontaktach
	Sympatia
	Zniecierpliwienie
	Obojętność

<table>
<tr><td rowspan="2"></td><td colspan="2">Współczucie</td></tr>
<tr><td colspan="2">Chęć pomocy</td></tr>
<tr><td colspan="3">Pytania ogólne po wybraniu opcji "Tak" w pytaniu Q: Czy jest Pan(i) profesjonalist(k)ą ochrony zdrowia?</td></tr>
<tr><td rowspan="11">Jaki zawód Pan(i) wykonuje?</td><td colspan="2">Dietetyk</td></tr>
<tr><td colspan="2">Opiekun medyczny</td></tr>
<tr><td colspan="2">Pielęgniarz/pielęgniarka</td></tr>
<tr><td colspan="2">Ratownik Medyczny</td></tr>
<tr><td colspan="2">Farmaceuta</td></tr>
<tr><td rowspan="3">Lekarz, jeśli tak to jakiej specjalności</td><td>Zabiegowy</td></tr>
<tr><td>Niezabiegowy</td></tr>
<tr><td>Medycyna rodzinna</td></tr>
<tr><td></td><td></td></tr>
<tr><td colspan="2">Fizjoterapeuta</td></tr>
<tr><td colspan="2">Inny niż wymieniony</td></tr>
<tr><td>Ile lat pracuje Pan(i) w zawodzie?</td><td colspan="2"></td></tr>
<tr><td rowspan="4">Pani/Pana główne miejsce pracy to:</td><td colspan="2">Szpital</td></tr>
<tr><td colspan="2">Poradnia specjalistyczna</td></tr>
<tr><td colspan="2">Podstawowa opieka zdrowotna</td></tr>
<tr><td colspan="2">Prywatna praktyka</td></tr>
</table>

<table>
<tr><td rowspan="3">Czy w swojej pracy ma Pan(i) do czynienia z pacjentami z otyłością?</td><td colspan="2">Tak, często</td></tr>
<tr><td colspan="2">Tak, ale rzadko</td></tr>
<tr><td colspan="2">Nie</td></tr>
<tr><td rowspan="10">Jakich odczuć doznaje Pan(i) podczas kontaktu z osobą z otyłością?</td><td colspan="2">Litość</td></tr>
<tr><td colspan="2">Niechęć</td></tr>
<tr><td colspan="2">Pogarda</td></tr>
<tr><td colspan="2">Życzliwość</td></tr>
<tr><td colspan="2">Dyskomfort w kontaktach</td></tr>
<tr><td colspan="2">Sympatia</td></tr>
<tr><td colspan="2">Zniecierpliwienie</td></tr>
<tr><td colspan="2">Obojętność</td></tr>
<tr><td colspan="2">Współczucie</td></tr>
<tr><td colspan="2">Chęć pomocy</td></tr>
<tr><td rowspan="2">Czy w Pana(i) ocenie opieka nad pacjentem z otyłością jest bardziej wymagająca niż nad pacjentem z prawidłową masa ciała (np. trudniej wykonać procedury medyczne, występują trudności techniczne podczas badania Pacjenta/opieki nad Pacjentem)?</td><td colspan="2">Tak</td></tr>
<tr><td colspan="2">Nie</td></tr>
<tr><td colspan="3">Pytania do każdego respondenta</td></tr>
<tr><td colspan="3">Skala Fatfobii</td></tr>
<tr><td colspan="3">Poniżej wymieniono 14 par przymiotników czasami wykorzystywanych do opisywania osób z otyłością. Dla każdego przymiotnika, dopasuj cyfrę od 1-5 najlepiej opisującą Twoje uczucia i przekonania.</td></tr>
<tr><td>Leniwa</td><td>1 – 2 – 3 – 4 - 5</td><td>Pracowita</td></tr>
</table>

Brak silnej woli	1 – 2 – 3 – 4 – 5	Ma silną wolę
Atrakcyjna	1 – 2 – 3 – 4 – 5	Nieatrakcyjna
Dobra samokontrola	1 – 2 – 3 – 4 – 5	Kiepska samokontrola
Szybka	1 – 2 – 3 – 4 – 5	Wolna
Mająca wytrwałośc	1 – 2 – 3 – 4 – 5	Nie mająca wytrwałości
Aktywna	1 – 2 – 3 – 4 – 5	Nieaktywna
Słaba	1 – 2 – 3 – 4 – 5	Silna
Lubiąca sobie dogadzać	1 – 2 – 3 – 4 – 5	Skłonna do poświęceń
Nielubiąca jedzenia	1 – 2 – 3 – 4 – 5	Lubiąca jedzenie
Nieforemna	1 – 2 – 3 – 4 – 5	Kształtna
Nie dojada	1 – 2 – 3 – 4 – 5	Je za dużo
Niepewna (siebie)	1 – 2 – 3 – 4 – 5	Pewna (siebie)
Niska samoocena	1 – 2 – 3 – 4 - 5	Wysoka samoocena

Pytania oceniające poziom stygmatyzacji.	
Czy w Pana(i) ocenie osoby z otyłością są gorsze od osób o prawidłowej wadze?	Zdecydowanie tak
	Tak
	Raczej tak
	Raczej nie
	Nie
	Zdecydowanie nie

Czy w Pana(i) ocenie osoby z otyłością są mniej atrakcyjne?	Zdecydowanie tak
	Tak
	Raczej tak
	Raczej nie
	Nie
	Zdecydowanie nie
Czy jako pracodawca zatrudnił(a)by Pan(i) osobę z otyłością?	Zdecydowanie tak
	Tak
	Raczej tak
	Raczej nie
	Nie
	Zdecydowanie nie
Czy w Pana(i) ocenie otyłość jest powodem do wstydu?	Zdecydowanie tak
	Tak
	Raczej tak
	Raczej nie
	Nie
	Zdecydowanie nie
W skali od 1-10 proszę zaznaczyć z jakim prawdopodobieństwem zatrudnił(a)by Pan(i) osobę z otyłością?	

W skali od 1-10 proszę zaznaczyć z jakim prawdopodobieństwem umówił(a)by się Pan(i) na randkę z osobą z otyłością?	
W skali od 1-10 proszę zaznaczyć z jakim prawdopodobieństwem powierzył(a)by Pan(i) pod opiekę swoje dzieci osobie z otyłością?	
W skali od 1-10 proszę zaznaczyć z jakim prawdopodobieństwem zaprzyjaźnił(a)by się Pan(i) z osobą z otyłością?	
W skali od 1 do 10 na ile ocenia Pan(i), że osoby z otyłością są dyskryminowane w społeczeństwie?	
Pytania dotyczące poziomu wiedzy na temat otyłości	
W Pana(i) opinii, otyłość:	jest chorobą przewlekłą, która wymaga leczenia
	jest chorobą, która nie wymaga leczenia
	nie jest choroba przewlekłą
Kryterium rozpoznania otyłości wykorzystując wskaźnik BMI (body mass index) obejmuje:	≥ 25
	≥ 27.5
	≥ 30
	≥ 29
Co w Pana(i) ocenie może być przyczyną otyłości (możliwa więcej niż jedna odpowiedź)	brak aktywności fizycznej
	nadmierna podaż kalorii
	choroby przewlekłe np. cukrzyca,
	nadczynność tarczycy

	działanie niektórych leków (np. stereoidów, leków przeciwpsychotycznych)
Skuteczną formą leczenia otyłości jest (możliwość wybrania więcej niż jednej):	leczenie operacyjne
	leczenie farmakologiczne
	regularna aktywność fizyczna
	głodówka
Czy Pana(i) zdaniem otyłość może być przyczyną (możliwość wybrania więcej niż jednej):	gorszej tolerancji wysiłku/szybszej męczliwości
	nadciśnienia tętniczego
	cukrzycy
	zaburzeń hormonalnych
	zaburzeń miesiączkowania u kobiet
	spadku libido
	pogorszenia kondycji włosów i/lub paznokci
	depresji
	demencji w przyszłości
	dny moczanowej
